# Roles of CpcF and CpcG1 in Peroxiredoxin-Mediated Oxidative Stress Responses and Cellular Fitness in the Cyanobacterium *Synechocystis* sp. PCC 6803

**DOI:** 10.3389/fmicb.2019.01059

**Published:** 2019-05-09

**Authors:** Sookyung Oh, Beronda L. Montgomery

**Affiliations:** ^1^MSU-DOE Plant Research Laboratory, College of Natural Science, Michigan State University, East Lansing, MI, United States; ^2^Department of Biochemistry and Molecular Biology, Michigan State University, East Lansing, MI, United States; ^3^Department of Microbiology and Molecular Genetics, Michigan State University, East Lansing, MI, United States

**Keywords:** cyanobacteria, oxidative stress, reactive oxgen species, peroxiredoxin, phycobilisome

## Abstract

As a component of the photosynthetic apparatus in cyanobacteria, the phycobilisome (PBS) plays an important role in harvesting and transferring light energy to the core photosynthetic reaction centers. The size, composition (phycobiliprotein and chromophore), and assembly of PBSs can be dynamic to cope with tuning photosynthesis and associated cellular fitness in variable light environments. Here, we explore the role of PBS-related stress responses by analyzing deletion mutants of *cpcF* or *cpcG1* genes in *Synechocystis* sp. PCC 6803. The *cpcF* gene encodes a lyase that links the phycocyanobilin (PCB) chromophore to the alpha subunit of phycocyanin (PC), a central phycobiliprotein (PBP) in PBSs. Deletion of *cpcF* (i.e., Δ*cpcF* strain) resulted in slow growth, reduced greening, elevated reactive oxygen species (ROS) levels, together with an elevated accumulation of a stress-related Peroxiredoxin protein (Sll1621). Additionally, Δ*cpcF* exhibited reduced sensitivity to a photosynthesis-related stress inducer, methyl viologen (MV), which disrupts electron transfer. The *cpcG1* gene encodes a linker protein that serves to connect PC to the core PBP allophycocyanin. A deletion mutant of *cpcG1* (i.e.,Δ*cpcG1*) exhibited delayed growth, a defect in pigmentation, reduced accumulation of ROS, and insensitivity to MV treatment. By comparison, Δ*cpcF* and Δ*cpcG1* exhibited similarity in growth, pigmentation, and stress responses; yet, these strains showed distinct phenotypes for ROS accumulation, sensitivity to MV and Sll1621 accumulation. Our data emphasize an importance of the regulation of PBS structure in ROS-mediated stress responses that impact successful growth and development in cyanobacteria.

## Introduction

Phycobilisomes (PBSs) are abundant light-harvesting protein complexes, which comprise up to 60% of the total protein content of cyanobacteria (Singh et al., 2015). These complexes are composed of a core and multiple peripheral rods, which are made up of phycobiliproteins (PBPs), and linker proteins, which connect PBPs. PBPs function as accessory light-harvesting proteins in PBS complexes during light absorption for photosynthesis. These pigmented proteins consist of apoproteins with covalently attached bilin chromophores (or tetrapyrroles), which are connected by lyases via thioether linkage on a cysteine residue of PBPs. There are three major classes of PBPs in cyanobacteria, including allophycocyanin (AP; λmax 650–655 nm) in the core of PBSs, as well as phycocyanin (PC; λmax 610–620 nm) and phycoerythrin (PE; λmax 540–570 nm), the latter two of which are in the rods (Adir, 2008). Linker proteins are primarily non-chromophorylated and influence the structure of PBSs for proper light energy transfer to photosystems (Sidler, 1994). In cyanobacteria, two rod-core linker proteins, CpcG1 and CpcG2, are required for linkage of PC to AP (Kondo et al., 2005, 2007; Chang et al., 2015). L_CM_ is a core-membrane linker that connects PBSs to the reaction centers in thylakoid membranes (de Lorimier et al., 1990). The interactions of PBPs, chromophores, and linker proteins are important for absorption, transfer, and funneling of light energy into the two photosystems, PSI and PSII, on the stromal thylakoid membranes (Singh et al., 2015).

CpcE and CpcF function together as a heterodimeric lyase responsible for attaching the light-absorbing phycocyanobilin (PCB) chromophore to the α subunit of phycobiliprotein PC (Fairchild et al., 1992; Fairchild and Glazer, 1994). Given the lack of the lyase to attach PCB to α-PC, a Δ*cpcF* mutant in *Fremyella diplosiphon* is PC and PBS-deficient (Whitaker et al., 2009). In analyses to assay the functional impact of maintaining flexible size and/or PBP content of PBSs in *F. diplosiphon*, we conducted competition-based growth assays to measure the ability of the Δ*cpcF* mutant to persist relative to WT during growth in continuous or fluctuating high-intensity light (Agostoni et al., 2016). We determined that the Δ*cpcF* mutant is outcompeted in continuous sinusoidal light, whereas it competes relatively well against WT in short-term fluctuating light (FL) conditions (Agostoni et al., 2016). The ability of the Δ*cpcF* mutant to compete well in FL was associated with an apparent fitness cost to WT of responding to light-induced production of reactive oxygen species (ROS), which was not observed in the Δ*cpcF* mutant (Agostoni et al., 2016). One notable response in the Δ*cpcF* mutant was a reduced accumulation of the orange carotenoid protein (OCP), a protein which is involved in non-photochemical quenching (NPQ) in cyanobacteria and protection against oxidative stress (Sedoud et al., 2014). OCP both binds to the core of PBSs under high light stress to facilitate a dissipation of the absorption of excess light energy as heat, in order to avoid overexcitation of PBS and associated light-induced damage, as well as serves to quench singlet oxygen (Sedoud et al., 2014). The reduced accumulation of OCP in the Δ*cpcF* mutant of *F. diplosiphon*, which was observed under both continuous and fluctuating light conditions, implies that there may be potential feedback from cellular PBS levels to regulate OCP abundance (Agostoni et al., 2016).

CpcG is responsible for connecting PBS rods to the core (Kondo et al., 2005, 2007). Deletion of *cpcG1* and *cpcG2* in *Synechocystis* sp. PCC 6803 (hereafter *Synechocystis*) results in reduced accumulation of PC, slower growth, and a defect in photosynthetic performance (Kondo et al., 2005, 2007). In *Anabaena* sp. strain PCC 7120, electron microscopy image analysis indicated that deletion of *cpcG1* and *cpcG2* causes the depletion of PBS rod attachment and low densities of the core, indicating a necessary role of linker proteins in the stabilization of PBS cores (Chang et al., 2015). We hypothesized that the reduced OCP accumulation in *F. diplosiphon* may have been triggered by low PBS core densities (Agostoni et al., 2016). Notably, Harris et al. (2016) found a strong cross-linkage between CpcG1 and OCP proteins, among other crosslinks of CpcG1 to ApcB, ApcC, and CpcC, using liquid chromatography coupled to tandem mass spectrometry (LC/MS-MS), suggesting an important role of CpcG1 in OCP-mediated light energy dissipation during photoprotection.

Reduction of the size of light-harvesting antenna has been proposed to support an increased efficiency of photosynthesis due to a reduction of the loss of excitation energy, reduced cell shading, and increased light penetration in cultures (Perrine et al., 2012). For example, truncation of antenna complexes by the disruption of genes encoding PBS subunits, such as *cpcC1*, *cpcC2*, or core-membrane linker protein, *apcE*, in *Synechocystis*, results in a reduction of photosynthetic productivity under moderate light conditions (i.e., 50 μmol photons m^–2^ s^–1^), whereas the disruption of those genes results in increased biomass and photosynthetic efficiency in high light conditions (i.e., 800 μmol photons m^–2^ s^–1^) (Page et al., 2012; Joseph et al., 2014; Kirst et al., 2014). Additional insights into the potential cellular stress responses of such PBS-deficient strains have not been fully explored.

To assess whether the reduced OCP accumulation phenotype observed for a Δ*cpcF* mutant in *F. diplosiphon* is observed for other cyanobacterial strains and to test the potential fitness implications of the interaction between CpcG1 and OCP, we assessed PBS-deficient strains of *Synechocystis*. We created and assessed Δ*cpcF* and Δ*cpcG1* mutants in *Synechocystis*. We measured fitness as relative growth of the mutant strains compared to WT and assessed oxidative stress responses. These studies identified cellular stress responses associated with reduced PBS abundance, including identifying a peroxiredoxin as a downstream effector of CpcF-mediated oxidative stress responses.

## Results

### Deletion of *cpcF* or *cpcG1* in *Synechocystis* sp. PCC 6803

**FIGURE 1:**
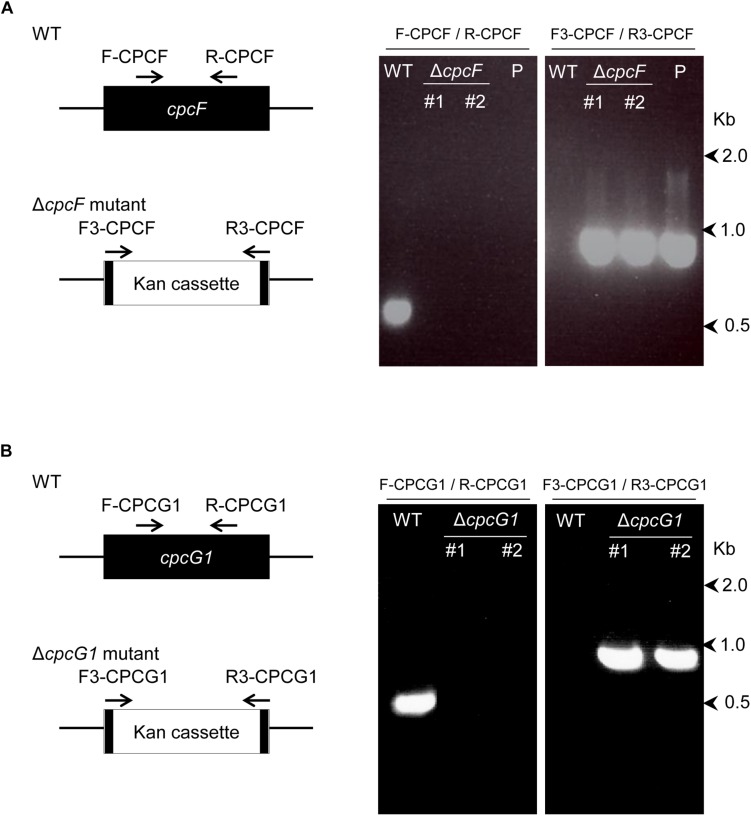
Deletion of *cpcF* gene **(A)** or *cpcG1* gene **(B)** in *Synechocystis* sp. PCC 6803. Each gene was replaced with kanamycin-resistance gene cassette by homologous recombination, utilizing PCR-driven overlap extension of 1 kb regions of upstream/downstream of gene and kanamycin gene. Deletion of each gene was confirmed by PCR amplification of each gene’s genomic region in the wild-type (WT) and deletion strains. Map of the position of the primers on the genes for the confirmation PCR was shown. Numbers to the right represent sizes in kilobases. Two independent deletion lines for each gene were used. P, control plasmid that was used for transformation.

We constructed Δ*cpcF* and Δ*cpcG1* deletion mutant strains via homologous recombination (Figure 1). Each gene from *Synechocystis* was replaced with a kanamycin-resistance gene via homologous recombination. Deletion of each gene was verified by genotyping using PCR amplification of each genomic region in the wild-type (WT) and deletion strains (Figure 1). PCR-based genotyping indicated a positive PCR amplification for the WT genes only in WT, whereas no signals were apparent in Δ*cpcF* and Δ*cpcG1* mutants (Figure 1). Analysis using kanamycin gene-specific primers resulted in a positive PCR amplification only in deletion mutants, but not in WT (Figure 1).

**FIGURE 2:**
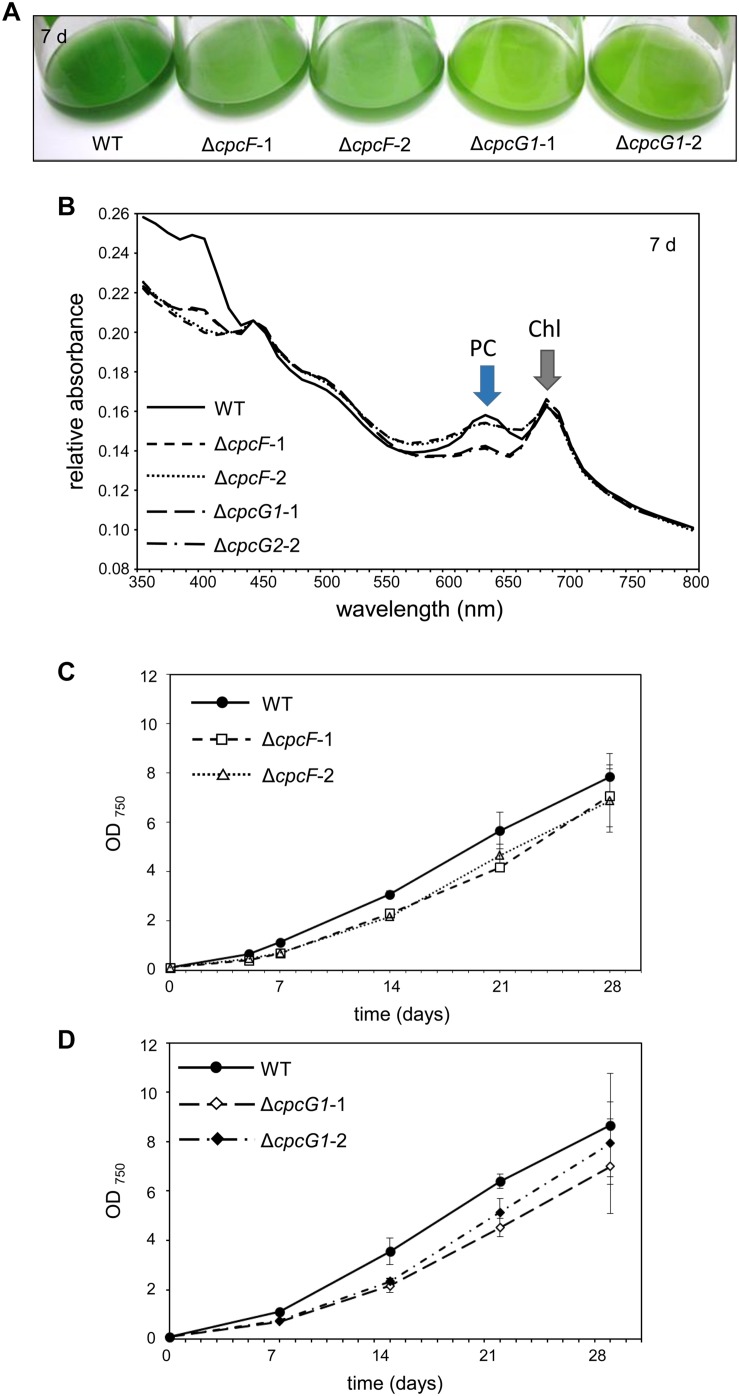
Growth and spectral scans of Δ*cpcF* or Δ*cpcG1* mutants compared to wild-type (WT) grown under 10 μmol m^–2^ s^–1^ of white light in BG-11/HEPES medium. **(A)** Liquid culture of the cells grown for 7 days. **(B)** Representative whole-cell absorbance spectral scans of WT, Δ*cpcF*, or Δ*cpcG1* mutant strains. PC, phycocyanin peak; Chl, chlorophyll peak. **(C,D)** Representative growth curves of wild-type (WT), Δ*cpcF*, and Δ*cpcG1* mutant strains over time. Cell growth was measured by optical density at 750 nm (OD_750_). Two independent deletion lines for each gene were used.

Δ*cpcF* or Δ*cpcG1* mutants exhibited reductions in blue-green coloration compared to WT when grown under low intensity white light (WL; 10 μmol m^–2^ s^–1^) in BG-11/HEPES medium (Figure 2A). Both Δ*cpcF* or Δ*cpcG1* mutants also exhibited reduced PC levels as expected (Figure 2B; compare height of PC peak to Chl peak). Both mutants also showed a defect in growth compared to WT (Figures 2C,D). Reduced growth relative to WT has been reported previously for Δ*cpcF* (Zhang et al., 2014) and Δ*cpcG1* (Kondo et al., 2005, 2007) in *Synechocystis* and other cyanobacterial strains.

**FIGURE 3:**
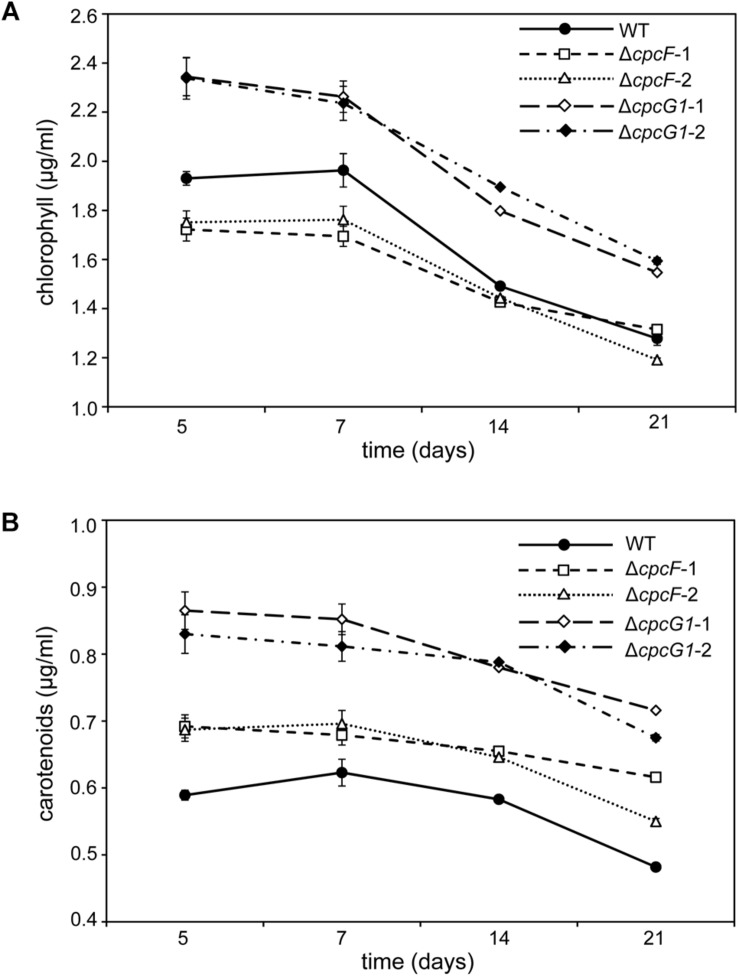
Photosynthetic pigments from WT, Δ*cpcF*, or Δ*cpcG1* mutant strains grown under 10 μmol m^–2^ s^–1^ of white light in BG-11/HEPES medium. Measurement of **(A)** chlorophyll *a* and **(B)** carotenoids were determined and representative data are shown with data points in line graphs representing means (± SD). Two independent deletion lines for each gene were used.

The level of chlorophyll in the Δ*cpcF* mutant was slightly lower than WT at early time points during time-course experiments, whereas the level of chlorophyll in ΔcpcG1 was higher than WT throughout the analyses (Figure 3A). We also measured the concentrations of carotenoids, which are light-harvesting pigments associated with the photosynthetic apparatus, and important photoprotective molecules for preventing photooxidative damage. We observed increased levels of carotenoids for both mutant strains with Δ*cpcG1* having a higher carotenoid level than both *cpcF* and WT (Figure 3B). Notably, a *F. diplosiphon* Δ*cpcF* mutant exhibits reduced chlorophyll, but no significant difference in carotenoid levels under high-light growth (Agostoni et al., 2016). Collectively, our data indicated the potential importance of both of these proteins in pigment-mediated photoprotection, yet provided evidence of distinct impacts for CpcF and CpcG1 on the accumulation of chlorophyll and carotenoids.

### Effects of Methyl Viologen (MV) on Δ*cpcF* or Δ*cpcG1* Mutants

**FIGURE 4:**
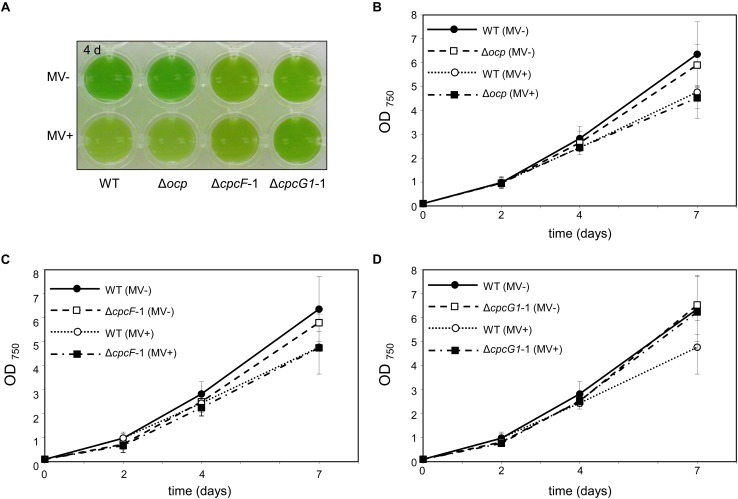
Impact of the treatment of methyl viologen (MV) on wild-type (WT), Δ*ocp*, Δ*cpcF*, or Δ*cpcG1* strains. All cyanobacterial strains were grown under 40 μmol m^–2^ s^–1^ of white light in BG-11/HEPES medium supplemented without (–) or with (+) 1 μM of MV. **(A)** Liquid culture of the cells grown for 4 days. **(B–D)** Growth curve of WT, Δ*ocp*, Δ*cpcF*, or Δ*cpcG1* strains over time. Cell growth was measured by optical density at 750 nm (OD_750_). Data points in line graphs represent means (± SD) of two biologically independent samples.

To probe the impact of oxidative stress on the growth of PBS-deficient strains and OCP levels, we treated cells with methyl viologen (MV) (Fujii et al., 1990). MV is a well-known herbicide and it acts as an artificial electron acceptor from photosystems, resulting in a disruption of the electron transport activity, ROS generation, and oxidative stress (Kim and Lee, 2002). Here, we tested the effect of MV on Δ*cpcF* and Δ*cpcG1* strains. In these analyses, we included the *ocp* deletion mutant, i.e., Δ*ocp*, which lacks OCP that is responsible for photoprotection through NPQ. MV treatment resulted in a reduction of chl levels (Figure 4A) and slowed growth (Figures 4B–D) in WT, as anticipated (Kobayashi et al., 2004; Ke et al., 2014). The Δ*ocp* mutant grew similar to WT independent of MV treatment (Figure 4B). The similarity in appearance between WT and Δ*ocp* in response to MV treatment was reminiscent of a lack of an observed difference between WT and a Δ*ocp* mutant in electron transport activity, measured by the uptake of oxygen during photosynthesis (Kusama et al., 2015). Neither Δ*cpcF* nor Δ*cpcG1* cultures exhibited a visible color change during MV treatment, indicating a potential resistance to MV treatment (Figure 4A). However, similar to WT, growth of Δ*cpcF* was reduced in the presence of MV, although Δ*cpcF* grew slower than WT throughout the analysis (Figure 4C). Notably, MV had a limited impact on the growth of the Δ*cpcG1* strain (Figure 4D).

### ROS Accumulation in Δ*cpcF* or Δ*cpcG1* Mutants

**FIGURE 5:**
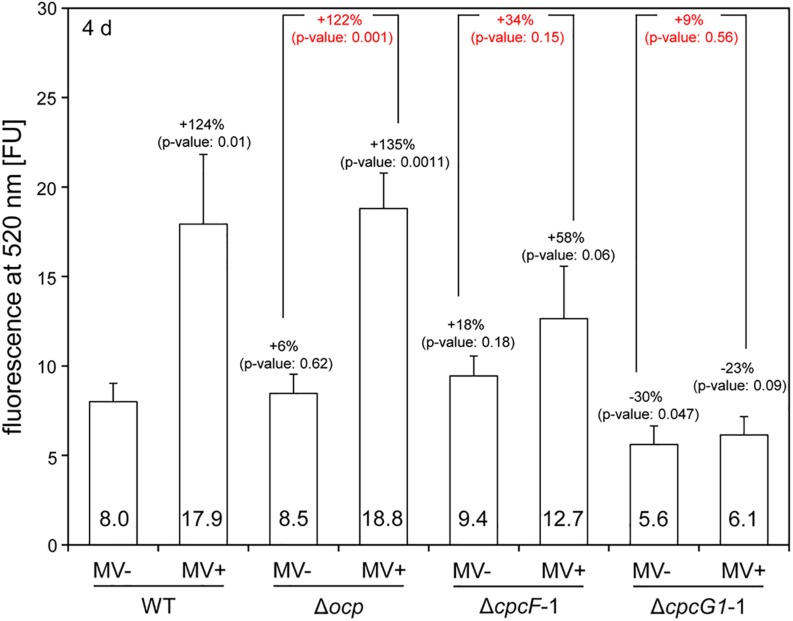
Accumulation of reactive oxygen species (ROS) in wild-type (WT), Δ*ocp*, Δ*cpcF*, or Δ*cpcG1* strains treated with methyl viologen (MV). The level of ROS was estimated by DCF-dependent fluorescence at 520 nm from the cells grown for 4 days under 40 μmol m^–2^ s^–1^ of white light in BG-11/HEPES medium supplemented without (–) or with (+) 1 μM of MV. Bars represent mean (± SD; *n* = 3). In black letters, percentages (number above bars) of change in ROS level relative to WT without MV treatment (WT, MV-), and *p*-values from unpaired, two-tailed Student’s *t*-test comparing strains with MV- or MV+ to WT were shown. In red letters, percentages (number above bars) of change in ROS level from MV+ relative to MV- for each mutant strain, and *p*-values from unpaired, two-tailed Student’s *t*-test comparing MV- and MV+ on each strain were shown.

Given the noted impact of MV on induction of ROS accumulation (Kim and Lee, 2002), we measured ROS levels in MV-treated cells using the ROS-sensitive dichlorodihydrofluorescein diacetate (DCFH-DA) dye (He and Häder, 2002). Similar to WT *F. diplosiphon* cells (Busch and Montgomery, 2015), WT *Synechocystis* exhibited significantly elevated ROS levels after MV treatment (Figure 5). The pattern of ROS accumulation after MV treatment in the Δ*ocp* mutant was similar to MV-treated WT (Figure 5). MV-dependent ROS accumulation in the Δ*cpcF* mutant was mild and not statistically significant compared to WT (Figure 5). Notably, ROS levels were significantly lower in the Δ*cpcG1* mutant before MV treatment compared to WT, and MV treatment did not contribute significantly to ROS accumulation in this mutant (Figure 5).

### Identification of Peroxiredoxins in Δ*cpcF*

**FIGURE 6:**
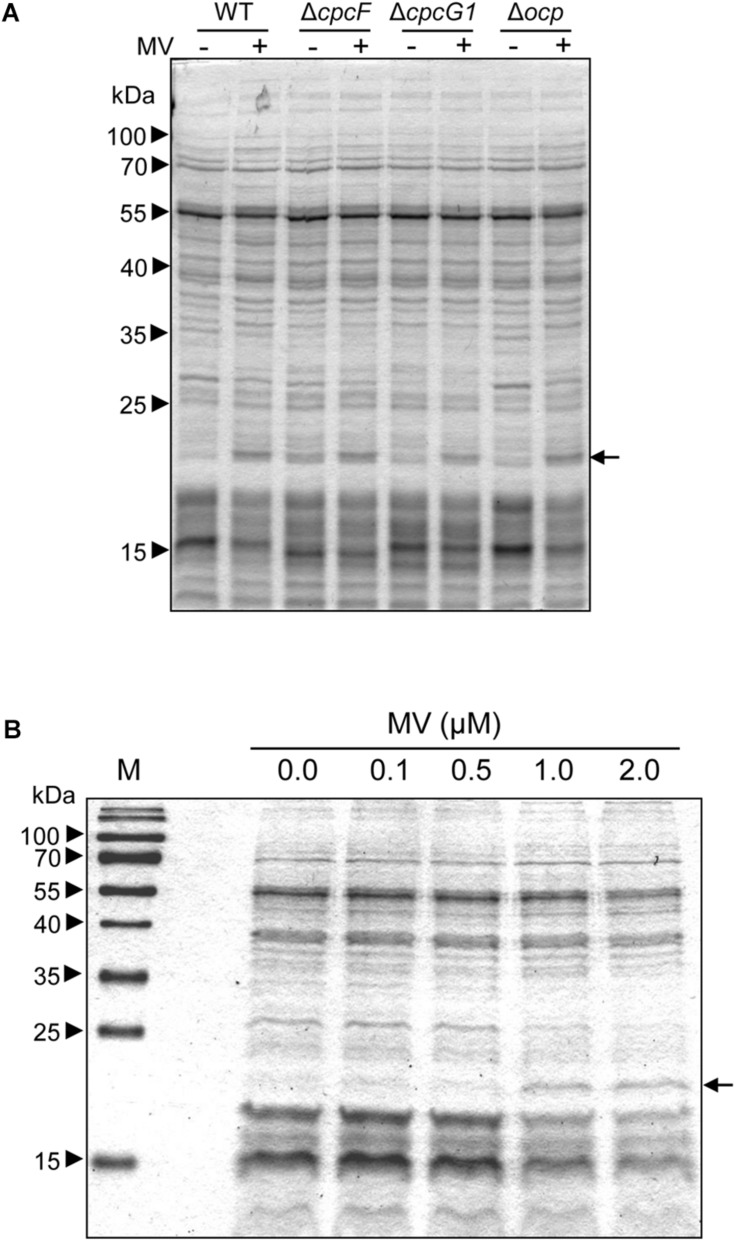
MV-dependent accumulation of proteins in *Synechocystis*. **(A)** MV-dependent protein accumulation in wild-type (WT), Δ*ocp*, Δ*cpcF*, or Δ*cpcG1* strains. 5 μg of total soluble protein from strains grown for 4 days were resolved on 12% (w/v) polyacrylamide gels by SDS-PAGE electrophoresis. A representative gel is shown. Arrowheads indicate relative size based on molecular weight markers; arrow indicates protein bands excised from gels for LC-MS/MS analysis. **(B)** Treatment of WT with various concentrations of MV. Total soluble proteins were extracted from wild-type strain grown under 40 μmol m^–2^ s^–1^ of white light in BG-11/HEPES medium supplemented with 0.0 to 2.0 μM of MV for 2 days. 2.5 μg of proteins were resolved on 15% polyacrylamide gel by SDS-PAGE electrophoresis. An arrow indicated protein bands excised from the gel for LC-MS/MS analysis.

To identify proteins related to MV responses in Δ*cpcF* or Δ*cpcG1* mutants, we surveyed differentially accumulated proteins after MV treatment. SDS-PAGE gel analyses showed that a distinctive 21 kDa protein band accumulated in WT, Δ*cpcG1*, or Δ*ocp* strains after MV treatment, whereas the 21 kDa protein accumulated to higher levels in Δ*cpcF* even prior to MV treatment (Figure 6A). To investigate whether the protein accumulation is associated with the degree of oxidative stress levels, we treated WT with various concentrations of MV and found higher accumulation of the 21 kDa protein band in the cells treated with increasing concentrations of MV (Figure 6B). For protein identification, we excised the 21 kDa-band and subjected it to LC-MS/MS analyses. We identified the MV-induced protein as a peroxiredoxin (Sll1621) (Table 1). Peroxiredoxins play an antioxidant role by catalyzing the reduction of various hydroperoxides (Dietz, 2003). Next, we tested the 21 kDa band from SDS-PAGE gels of proteins isolated from WT and Δ*cpcF* and found that the Sll1621 peroxiredoxin protein was indeed the protein induced in WT by MV and which highly accumulated in the Δ*cpcF* mutant prior to MV treatment (Table 2).

**TABLE 1:** Identification of peroxiredoxin proteins differentially accumulated in wild-type under the treatment of various concentration of MV using liquid chromatography tandem mass spectrometry (LC-MS/MS) analysis.

**Name**	**ID**	**MV**	**Normalized total spectra**				
			**MV (μM)**				
			**0.0**	**0.1**	**0.5**	**1.0**	**2.0**
Peroxiredoxin	Sll1621	21 kDa	3	3	5	8	6
Trypsin from PIG	N/A	24 kDa	5	6	4	4	5
50S ribosomal protein L6	RplF	20 kDa	3	3	4	2	N/D
Inorganic pyrophosphatase	Ppa	19 kDa	1	1	4	1	2

*Proteins listed were identified with probabilities of ≥95% using Scaffold software (Proteome Software, Inc.) after spectral searches with the Mascot database search engine (Matrix Science, Inc.). N/A, not available; N/D, not detected.*

**TABLE 2:** Identification of peroxiredoxin proteins differentially accumulated in wild-type (WT) and Δ*cpcF* mutant using liquid chromatography tandem mass spectrometry (LC-MS/MS) analysis.

**Name**	**ID**	**MW**	**Normalized total spectra**			
			**WT**		**Δ*cpcF-*1**	
			**MV−**	**MV+**	**MV−**	**MV+**
Peroxiredoxin	Sll1621	21 kDa	7	12	10	11
50S ribosomal protein L6	RplF	20 kDa	N/D	1	N/D	1
Inorganic pyrophosphatase	Ppa	19 kDa	N/D	1	N/D	1

*Proteins listed were identified with probabilities of ≥95 % using Scaffold software (Proteome Software, Inc) after spectral searches with the Mascot database search engine (Matrix Science, Inc.). N/D, not detected.*

### OCP Protein Accumulation in PBS-Deficient Strains

**FIGURE 7:**
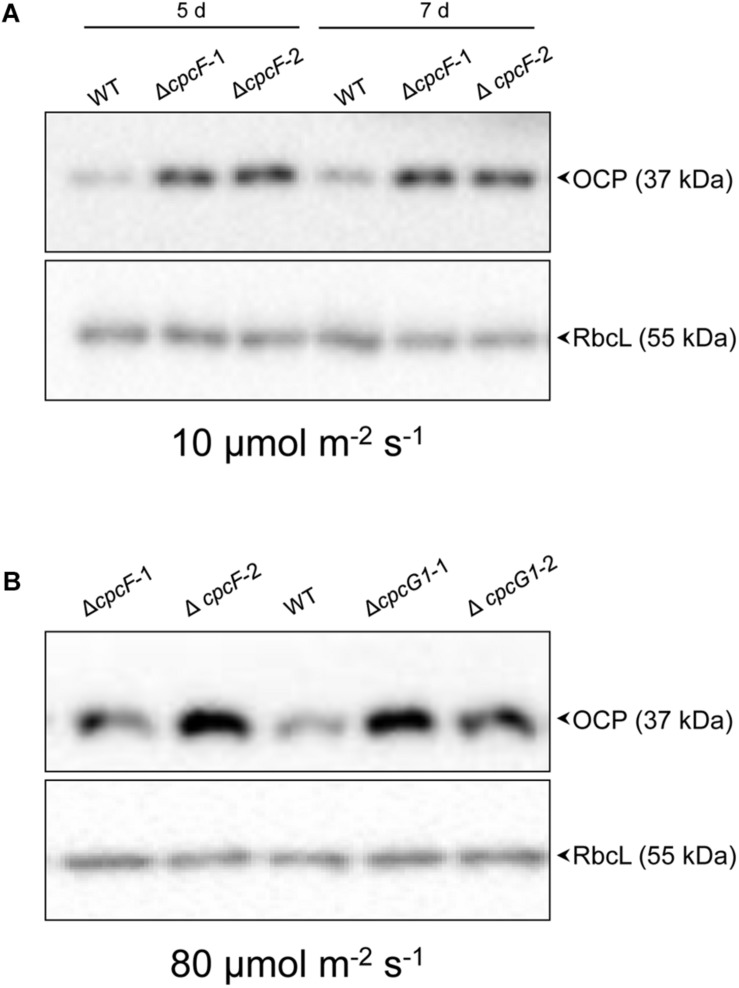
Accumulation of OCP protein in WT and phycobilisome-deficient strains. Immunoblot analysis was performed using anti-OCP antibody (top panels) or anti-RbcL antibody (bottom panels), with representative blots shown. Total soluble proteins were extracted from wild-type (WT), Δ*cpcF* and Δ*cpcG1* strains (two independent mutant strains for each Δ*cpcF* and Δ*cpcG1* included) grown under **(A)** 10 μmol m^–2^ s^–1^ for 5 or 7 days or **(B)** 80 μmol m^–2^ s^–1^ for 5 days of white light in BG-11/HEPES medium. 2.5 μg of proteins were resolved on 10% (w/v) polyacrylamide gels by SDS-PAGE electrophoresis prior to immunoblotting. Arrowheads indicated OCP or RbcL proteins.

Orange carotenoid protein is important for quenching excessive light energy and mitigating ROS in cyanobacteria (Sedoud et al., 2014). In *F. diplosiphon*, deletion of *cpcF* resulted in low accumulation of ROS and OCP proteins in stressful high light conditions (Agostoni et al., 2016). In *Synechocystis*, deletion of *cpcF* did not cause low accumulation of ROS (Figure 5). Relatedly, the Δ*cpcF* mutation caused a higher accumulation of OCP proteins in 10 μmol m^–2^ s^–1^ of white light (Figure 7A). Δ*cpcG1* did not have a difference in OCP accumulation in low light (Supplementary Figure S1).

**FIGURE 8:**
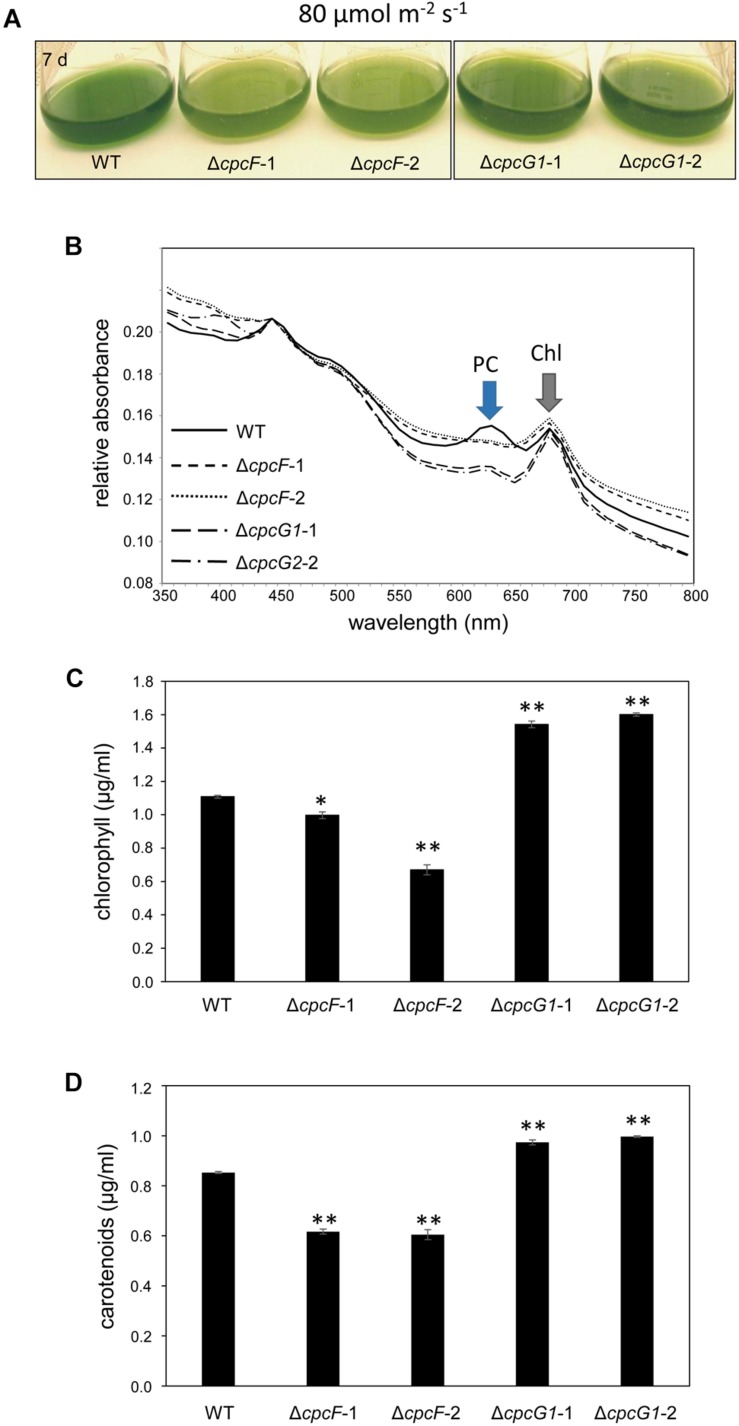
8 Growth, spectral scan and pigment analyses of Δ*cpcF* or Δ*cpcG1* mutants compared to wild-type (WT) grown under 80 μmol m^–2^ s^–1^ of white light in BG-11/HEPES medium. **(A)** Liquid culture of the cells grown for 7 days. **(B)** Representative whole-cell absorbance spectral scans of WT, Δ*cpcF*, or Δ*cpcG1* mutant strains. PC, phycocyanin peak; Chl, chlorophyll peak. Levels of **(C)** chlorophyll *a* and **(D)** carotenoids were determined and representative data are shown with bars representing means (± SD). Two independent deletion lines for each gene were used. Unpaired, two-tailed Student’s *t* test comparing strains with WT, *p < 0.05, ^∗∗^*p* < 0.001.

We also assessed OCP accumulation in Δ*cpcF* and Δ*cpcG1* strains under higher light intensity, i.e., 80 μmol m^–2^ s^–1^, to determine if more stressful light conditions differentially impacted the strains, especially OCP levels. Both Δ*cpcF* and Δ*cpcG1* exhibited reduced blue-green coloring and reduced PC levels under increased light intensity, with Δ*cpcF* having greater impairments (Figures 8A,B). Similar to low light conditions, Δ*cpcF* strains exhibited lower chl*a* levels than WT (compare Figures 3A, 8C). However, under increased light intensity the Δ*cpcF* strain exhibited a significant reduction in carotenoid levels (Figure 8D), rather than the elevated carotenoid levels apparent in this strain compared to WT under low white light (Figure 3B). Δ*cpcG1* exhibited significantly higher chlorophyll and carotenoid levels compared to WT independent of changes in light intensity under which the strain was grown (compare Figure 3 with Figures 8C,D). Under higher light intensity, OCP levels were elevated in Δ*cpcF* compared to WT (Figure 7B), similar to low light conditions. Under elevated light intensity, OCP levels were also higher in Δ*cpcG1* than WT, suggesting that in the Δ*cpcG1* strain misregulation of OCP accumulation is light-intensity-dependent.

Given the overaccumulation of OCP in PBS-deficient mutants of *Synechocystis* observed here in elevated light intensity compared to lower OCP accumulation in a Δ*cpcF* strain of *F. diplosiphon* under high light conditions (Agostoni et al., 2016), we also assessed OCP accumulation in the WT and Δ*cpcF* strains of *F. diplosiphon* under similar low light conditions used herein. OCP levels in the *F. diplosiophon* Δ*cpcF* mutant under low light conditions (i.e., 10 μmol m^–2^ s^–1^) were not substantially different from WT (Supplementary Figure S2). Thus, there are species-specific differences in the regulation of OCP levels in PBS-deficient strains.

## Discussion

In this study, we demonstrated the effect of deletion of *cpcF* and *cpcG1* genes in *Synechocystis* on growth, pigmentation, accumulation of a stress-related peroxiredoxin protein, and ROS production. CpcF is a subunit of a lyase complex, which functions to link PCB to α-PC, and the deletion of *cpcF* results in decreased PBS size (Swanson et al., 1992; Zhou et al., 1992; Zhang et al., 2014). CpcG1 connects rods to the core and deletion of the *cpcG1* gene is expected to yield cells containing only AP cores on the thylakoid membrane or destabilized cores (Kondo et al., 2005). A Δ*cpcF* mutant of *Synechococcus* sp. PCC 7002 exhibits reduced PC and chlorophyll levels, as well as reduced growth relative to WT (Zhou et al., 1992), similar to the phenotype we observed here for a *Synechocystis* Δ*cpcF* mutant. Additionally, we observed an even greater reduction of PC levels in a *Synechocystis* Δ*cpcG1* mutant, although the strain accumulated higher levels of chlorophyll than WT. This elevated chlorophyll in Δ*cpcG1* correlates with a previously observed increase in PSII levels in a CpcG1-deficient strain (Kondo et al., 2005).

Carotenoids have a role in anti-oxidative stress responses in many organisms, including cyanobacteria (Zhu et al., 2010) and plants (Havaux, 2014). Under low to moderate light conditions, we observed elevated levels of carotenoids and decreased sensitivity to MV in both Δ*cpcF* and Δ*cpcG1* mutants, although carotenoid levels and MV insensitivity were greater in the Δ*cpcG1* strain (Figures 3B, 5). Treatment with MV caused oxidative stress in WT as indicated by higher accumulation of ROS (Figure 5), which was consistent with reports of increased ROS levels in MV-treated *Anabaena* sp. PCC7120 and *F. diplosiphon* cells (Panda et al., 2014; Busch and Montgomery, 2015). As both Δ*cpcF* and Δ*cpcG1* mutants lack a response to MV in regards to ROS levels, the altered carotenoid content in the mutants may contribute to the mitigation of oxidative stress and MV resistance. Additionally, the reduced capacity for light absorption in PBS mutants could result in a reduction in the photosynthetic electron transport and, thus, these strains may not show as significant a response to MV as in WT under the conditions tested. Indeed, the Δ*cpcF* mutant harbors PBSs with extremely truncated rods and which are not completely functional in regards to excitation energy transfer (Zhang et al., 2014). By contrast, the Δ*cpcG1* strain cannot attach rods to the AP core and, thus, exhibits reduced energy transfer to PSII (Kondo et al., 2005). These distinctions in the photosynthetic apparatus may explain why MV still has some impact, although mitigated, in the Δ*cpcF* strain which has truncated PBS still attached to PSII, whereas Δ*cpcG1* lacks excitation of PSII and would be presumed to also have significantly reduced photosynthetic electron transfer.

In our experiments, MV caused a high accumulation of a stress-related protein, peroxiredoxin (Prx, Sll1621) in WT *Synechocystis* (Figure 6 and Table 1), which was consistent with rapid upregulation of the *sll1621* gene after MV treatment (Kobayashi et al., 2004). Peroxiredoxins (Prxs) are peroxidases used for the reduction of various types of hydroperoxides and can be grouped into four classes depending on the composition of subunits and cysteine residues (Dietz, 2003, 2011; Hosoya-Matsuda et al., 2005). Prx Sll1621 is a type II Prx, which utilizes thioredoxin (Trx) and glutaredoxin (Grx) as electron donors with two catalytic cysteine residues (Choi et al., 1999). Hosoya-Matsuda et al. (2005) demonstrated that recombinant Sll1621 protein can detoxify a broad range of peroxides including, H_2_O_2_, butyl hydroperoxide, and cumene hydroperoxide. Our Δ*cpcF* mutant exhibited high levels of Sll1621 and MV treatment failed to further increase accumulation of this protein in this strain (Figure 6 and Table 2). This response was distinct from the Δ*cpcG1* mutant and suggests that multiple mechanisms, including carotenoid and peroxiredoxin accumulation, may contribute to MV resistance in Δ*cpcF*.

Orange carotenoid protein is a carotenoid-associated protein that functions in photoprotection by releasing excessive absorbed light energy as heat (Wilson et al., 2006). In *F. diplosiphon*, deletion of *cpcF* causes downregulation of *ocp* and reduced accumulation of OCP protein under high light conditions, indicating a cellular mechanism for inhibition of OCP production in the absence of PBSs in this strain (Agostoni et al., 2016). However, in this current study of *Synechocystis*, OCP protein was highly accumulated in a Δ*cpcF* PBS-deficient mutant when compared with WT under low light, and in both Δ*cpcF* and Δ*cpcG1* under higher light conditions (Figure 7). Relatedly, a *Synechocystis* strain completely lacking PBS due to deletion of *apcAB* and *apcE* has greatly elevated accumulation of OCP and increased oxidative stress (Kwon et al., 2013), similar to the phenotypes for Δ*cpcF* observed here. Thus, the difference between cyanobacterial species could be due to strain-dependent distinctions. Indeed, when we assessed OCP accumulation in the Δ*cpcF* strain of *F. diplosiphon* compared to WT under low light conditions, the *F. diplosiphon* strain did not accumulate higher levels of OCP. These differences could be related to distinct *ocp* gene complements in distinct strains. For instance, in *Synechocystis*, there is only one copy of canonical OCP (*slr1963*), whereas in *F. diplosiphon*, there are two genes encoding full-length OCP, canonical *ocp1*, and recently identified non-canonical *ocp2* (Bao et al., 2017). Notably, a Δ*cpc* operon deletion strain for *Synechocystis* grows similar or faster than WT under high light conditions (Kirst et al., 2014), similar to what we observed previously for a Δ*cpcF* strain of *F. diplosiphon* (Agostoni et al., 2016). Nonetheless, PBS deficiency is not a general signal for downregulating OCP accumulation across the board in different species of cyanobacteria.

Herein, we emphasized an importance of size and assembly of PBS in the regulation of peroxiredoxin-associated stress responses by demonstrating the impact of deletion of *cpcF* or *cpcG1* in *Synechocystis*. CpcF, which catalyzes the ligation of chromophores to α-PC, was required for cell growth, pigmentation, MV responses, and regulation of peroxiredoxin accumulation. The Δ*cpcG1* mutant, which disrupts *cpcG1* that encodes a structural protein for linkage of PC to AP, in many ways exhibited a similar mutant phenotype as the Δ*cpcF* deletion mutant, with the exception of a significant change in the accumulation of a peroxiredoxin protein and greater reductions in PC.

## Materials and Methods

### Strains and Growth Conditions

We used *Synechocystis* sp. PCC 6803 as wild-type (WT). Strains were routinely grown in 20 mM HEPES-containing BG-11 medium (pH 8.0) at 28°C under 10 μmol m^–2^ s^–1^ white light illumination, unless a different light intensity is indicated. Light fluence rates were measured using a Li-Cor light meter (model LI-250, Li-Cor, Lincoln, NE, United States) with a connected Li-Cor quantum sensor (model LI-190SA).

### Deletion of *cpcF* or *cpcG1* Genes in *Synechocystis* sp. PCC 6803

Genomic DNA from *Synechocystis* sp. PCC 6803 was isolated using Quick-DNA Fungal/Bacterial Miniprep Kit (Cat. No. D6005, Zymo Research). Each gene was replaced with kanamycin-resistance gene (KanR) by homologous recombination, utilizing PCR-driven overlap extension of ∼1 kb regions of upstream/downstream of gene and KanR. In detail of the deletion of CPCF, F1-CPCF/R1-CPCF primers and F2-CPCF/R2-CPCF primers were used to generate PCR products for upstream and downstream of CPCF, and F3-CPCF/R3-CPCF primers were used to generate Kanamycin gene containing the flanking sequence of CPCF. The three PCR products used together as templates in amplification with primers F1-CPCF and R2-CPCF to generate the PCR fragment containing upstream-KanR-downstream of CPCF. For the deletion of CPCG1, F1-CPCG1/R1-CPCG1 primers and F2-CPCG1/R2-CPCG1 primers were used to generate PCR products for upstream and downstream of CPCG1, and F3-CPCG1/R3-CPCG1 primers were used to generate Kanamycin gene containing the flanking sequence of CPCG1. The three PCR products used together as templates in amplification with primers F1-CPCG1 and R2-CPCG1 to generate the PCR fragment containing upstream-KanR-downstream of CPCG1. PCR fragments were amplified with PrimeSTAR®Max DNA Polymerase (Cat. No. R045, Takara-Clontech), following a manufacturer’s instruction. The single, about 3 kb-long PCR fragment containing upstream-KanR-downstream of each gene was introduced in the pCR8/GW/TOPO vector (Invitrogen). Insert in the vector was validated by DNA-sequencing. 1 μg of the plasmid DNA was introduced into *Synechocystis* sp. PCC 6803 and transformants were found by test for kanamycin resistance (25 μg/ml) on BG-11 solid-medium. Deletion of each gene was confirmed by PCR analysis of each gene genomic region using gene specific primers (F-CPCF/R-CPCF, F-CPCG1/R-CPCG1) or upstream-downstream gene-specific primers as described in Figure 1. Primers used were listed in Supplementary Table S1.

### Measurement of Cell Growth, Absorption Spectrum, Pigments

Cell growth and absorption spectrum were measured as described in Whitaker et al. (2009).

Pigments were extracted and quantified as described (Tandeau de Marsac and Houmard, 1988; Kahn et al., 1997; Bordowitz and Montgomery, 2008) with a cell pellet of 1 ml culture that was adjusted at OD_750_ of 0.6.

### Treatment With Methyl Viologen (MV)

Wild-type (WT), Δ*ocp*, Δ*cpcF*, or Δ*cpcG1* mutants were grown under 40 μmol m^–2^ s^–1^ of white light at 28°C in BG-11/HEPES medium supplemented without (−) or with (+) of MV (Final concentration: 1 μM, Sigma). Before supplementing without (−) or with (+) MV, cells were adjusted to an OD_750_ of 0.1.

### Protein Extraction, SDS-PAGE, and Immunoblot Analysis

Total soluble protein was extracted from WT or mutant cells, adjusted to an OD_750_ of 0.5 using extraction buffer (50 mM Tris–HCl, pH 7.5, 0.5 mM PMSF) with 0.1 mm glass beads (Scientific Industries). Proteins were resolved on polyacrylamide gels by SDS-PAGE electrophoresis and immunoblotted to polyvinylidene difluoride (PVDF) membrane as described (Montgomery et al., 1999) with several modifications. The membrane was blocked with 2% (w/v) bovine serum albumin (BSA) in tris-buffered saline and incubated with 1:10000 dilution of anti-OCP antibody (Agostoni et al., 2016) or 1:3000 dilution of anti-RbcL antibody (Cat. No. AS07 218, Agrisera) followed by horseradish peroxidase-conjugated anti-rabbit IgG (Pierce 1858415; Lot No. HJ108849; 1:3000 diluted). Protein signal was developed using WesternBright enhanced chemiluminescence (Advansta) and collected using a ChemiDoc MP system (Bio-Rad).

### Measurement of Reactive Oxygen Species (ROS)

The level of ROS was estimated using a cell-permeable ROS-sensitive dye 2′, 7′ dichlorodihydrofluorescein diacetate (DCFH-DA, VWR) as described previously (Singh and Montgomery, 2012) with a few modifications. Before adding DCFH-DA, cell cultures were diluted to OD_750_ of 0.5 in 1 ml of BG-11/HEPES liquid medium and cells were incubated with DCFH-DA for 3 h at room temperature in the dark.

### Liquid Chromatography Tandem Mass Spectrometry (LC-MS/MS) Analysis

Total soluble proteins (10 μg for Table 1, 20 μg for Table 2) were resolved on 12% (w/v) polyacrylamide SDS-PAGE gels. The protein bands from WT and mutant samples in the gel were cut out and subjected to tryptic digestion followed by LC-MS/MS. Procedures for LC-MS/MS and database searching for homology to known peptides from *Synechocystis* sp. PCC 6803 were carried out at the Research Technology Support Facility (RTSF) at Michigan State University. For the matching of MS/MS spectra to peptide sequences, Mascot search engine (Matrix Science, Inc., Boston, MA, United States) was utilized. Proteins were identified with more than 95% of protein identification probability by Scaffold software (Proteome Software, Inc., Portland, OR, United States) and the normalized total Spectra were computed to compare samples.

## Data Availability

All datasets generated for this study are included in the manuscript and/or the Supplementary Files.

## Author Contributions

SO conceived and conducted experiments and wrote the manuscript. BM conceived experiments and wrote the manuscript.

## Conflict of Interest Statement

The authors declare that the research was conducted in the absence of any commercial or financial relationships that could be construed as a potential conflict of interest.
