# Supplementary material for: Roles of CpcF and CpcG1 in Peroxiredoxin-Mediated Oxidative Stress Responses and Cellular Fitness in the Cyanobacterium Synechocystis sp. PCC 6803
Source: Front Microbiol. 2019 May 9;10:1059. doi: 10.3389/fmicb.2019.01059 (PMC6521580; doi:10.3389/fmicb.2019.01059)
Supplement: Supplementary file 1 [file Data_Sheet_1.PDF]

Supplementary Material

Roles of CpcF and CpcG1 in Peroxiredoxin-Mediated Oxidative Stress Responses and Cellular Fitness in the Cyanobacterium *Synechocystis* sp. PCC 6803

Running Head: PBS regulation & cellular stress in cyanobacteria

Sookyung Oh[1] and Beronda L. Montgomery[1,2,3*]

[1]Department of Energy – Plant Research Laboratory, Michigan State University, East Lansing, MI 48824, USA

[2]Department of Biochemistry and Molecular Biology, Michigan State University, East Lansing, MI 48824, USA

[3]Department of Microbiology & Molecular Genetics, Michigan State University, East Lansing, MI 48824, USA.

*For correspondence: E-mail: montg133@msu.edu

Supplemental Table 1. Primer sequences

Primer ID	Primer sequence	Purpose
F1-*CPCF*	GTTTACCCTTTAGCCGCATTGAT	Deletion of cpcF
R1-*CPCF*	CCCGTTGAATATGGCTCATGGTTTAGCCCCTG	Deletion of cpcF
F2-*CPCF*	GATGCTCGATGAGTTTTTCTAAGCTTTTAGAGCACCTC	Deletion of cpcF
R2-*CPCF*	GGGAGCCAAGCTAGAATCAATG	Deletion of cpcF
F3-*CPCF*	CAGGGGCTAAACCATGAGCCATATTCAACGGG	Deletion of cpcF, Confirmation of deletion of cpcF
R3-*CPCF*	GAGGTGCTCTAAAAGCTTAGAAAAACTCATCGAGCATC	Deletion of cpcF, Confirmation of deletion of cpcF
F-*CPCF*	TCTTGTTCTTCCTGGGGTAGG	Confirmation of deletion of cpcF
R-*CPCF*	AGACTCGGCAGCCAAATTAGT	Confirmation of deletion of cpcF
F1-*CPCG1*	GATTTTTATTACCTGCTGGGGGAAG	Deletion of cpcG1
R1-*CPCG1*	CCCGTTGAATATGGCTCATGTGTAAACCTCCG	Deletion of cpcG1
F2-*CPCG1*	GATGCTCGATGAGTTTTTCTAAGCACTAAGGTCAGAG	Deletion of cpcG1
R2-*CPCG1*	GCCAAACAACGCCATAATCACTAA	Deletion of cpcG1
F3-*CPCG1*	CGGAGGTTTACACATGAGCCATATTCAACGGG	Deletion of cpcG1, Confirmation of deletion of cpcG1
R3-*CPCG1*	CTCTGACCTTAGTGCTTAGAAAAACTCATCGAGCATC	Deletion of cpcG1, Confirmation of deletion of cpcG1
F-*CPCG1*	CTCCAGCAGCGATATGGATAA	Confirmation of deletion of cpcG1
R-*CPCG1*	GTACCGGGGAGATTTGATGTT	Confirmation of deletion of cpcG1

Supplemental Figure 1. Accumulation of OCP protein in WT and phycobilisome-deficient Δ*cpcF* and Δ*cpcG1* strains. Immunoblot analysis was performed using anti-OCP antibody (top panel) or anti-RbcL antibody (bottom panel), with representative blots shown. Total soluble proteins were extracted from wild-type (WT), Δ*cpcF* and Δ*cpcG1* strains (two independent mutant strains for each Δ*cpcF* and Δ*cpcG1* included, as well as a Δ*cpcG1* strain that is segregating [seg.] a WT *cpcG1* gene and, thus, is similar to WT) grown under 10 µmol m^{-2} s^{-1} for 5 days or 7 days of white light in BG-11/HEPES medium. 2.5 µg of proteins were resolved on 10 % (w/v) polyacrylamide gels by SDS-PAGE electrophoresis prior to immunoblotting. Arrowheads indicated OCP or RbcL proteins.

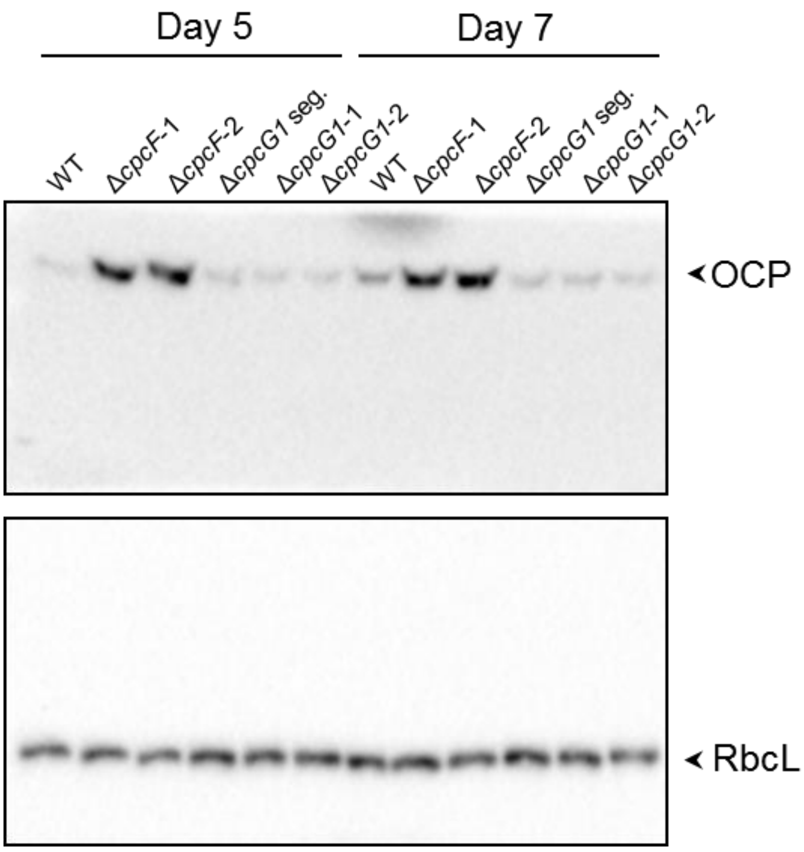

Supplemental Figure 2. Growth of *Fremyella diplosiphon* Δ*cpcF* mutants compared to wild-type (WT) and comparative accumulation of OCP protein in *Synechocystis* and *F. diplosiphon* Δ*cpcF* mutants and WT. (A) Liquid culture of the cells grown under 10 µmol m^{-2} s^{-1} of white light for six days. (B) *F. diplosiphon* WT and Δ*cpcF* strains were grown under 10 µmol m^{-2} s^{-1} of white light at 28 ºC in BG-11/HEPES medium for 3 days. Soluble proteins were extracted using CelLytic B Bacterial Cell Lysis/Extraction reagent (Sigma) with 30 mg of glass beads (Sigma) as previously described (Agostoni et al., 2013). Immunoblot analysis was performed using anti-OCP antibody (top panel; 1:1000 dilution) or anti-RbcL antibody (bottom panel; 1:5000 dilution), with representative blots shown. 2.5 µg of *Synechocystis* and 30 µg of *F. diplosiphon* proteins were resolved on 12% (OCP) or 10% (RbcL) polyacrylamide gels by SDS-PAGE electrophoresis prior to immunoblotting. Arrowheads indicated OCP or RbcL proteins.

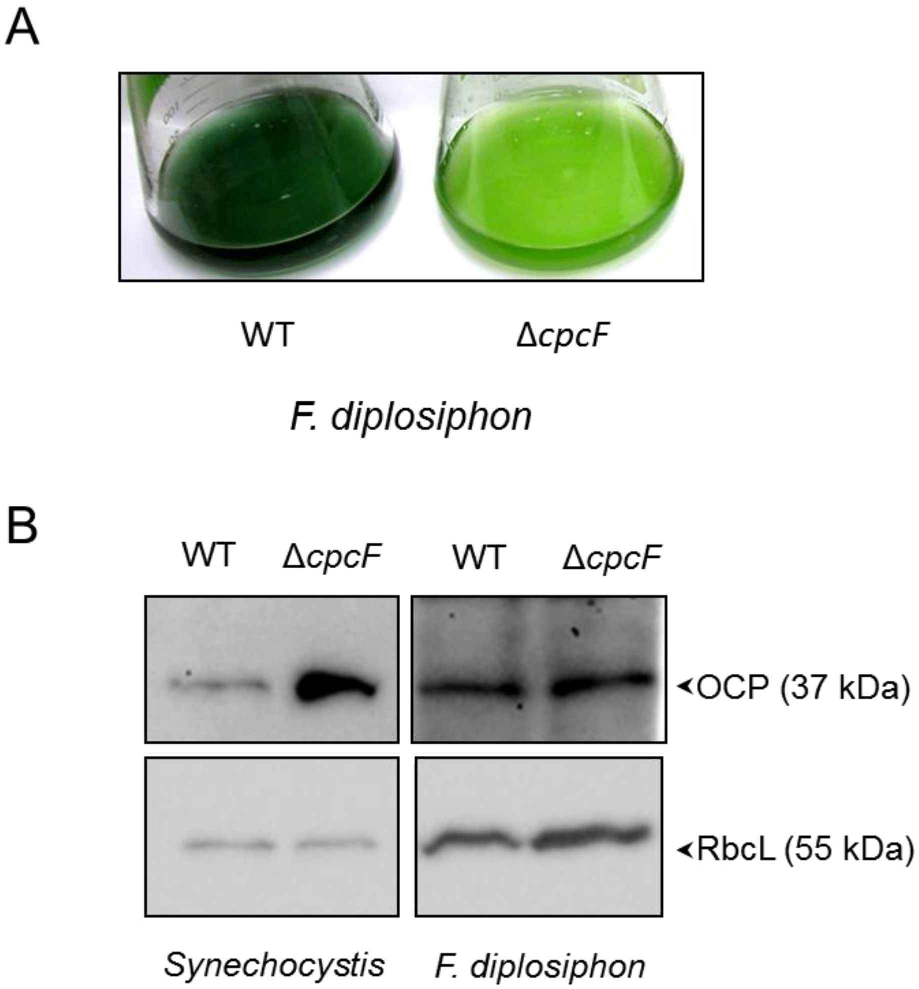

REFERENCES

Agostoni, M., Koestler, B. J., Waters, C. M., Williams, B. L., and Montgomery, B. L. (2013). Occurrence of cyclic di-GMP-modulating output domains in cyanobacteria: An illuminating perspective. *mBio* 4, e00451-13.
